# Rationale and design of the Multicenter Medication Reconciliation Quality Improvement Study (MARQUIS)

**DOI:** 10.1186/1472-6963-13-230

**Published:** 2013-06-25

**Authors:** Amanda H Salanitro, Sunil Kripalani, JoAnne Resnic, Stephanie K Mueller, Tosha B Wetterneck, Katherine Taylor Haynes, Jason Stein, Peter J Kaboli, Stephanie Labonville, Edward Etchells, Daniel J Cobaugh, David Hanson, Jeffrey L Greenwald, Mark V Williams, Jeffrey L Schnipper

**Affiliations:** 1 Geriatric Research, Education and Clinical Center, VA Tennessee Valley Healthcare System, Nashville, TN, USA; 2 Section of Hospital Medicine, Division of General Internal Medicine and Public Health, Department of Medicine, Vanderbilt University, Nashville, TN, USA; 3 Center for Hospital Innovation and Improvement, Society of Hospital Medicine, Philadelphia, PA, USA; 4 Division of General Medicine, Brigham and Women’s Hospital and Harvard Medical School, Boston, MA, USA; 5 Center for Quality and Productivity Improvement, UW Madison; Department of Medicine, University of Wisconsin School of Medicine and Public Health, Madison, Wisconsin, USA; 6 Peabody College, Vanderbilt University, Nashville, TN, USA; 7 Department of Medicine Quality Program, Emory University, Atlanta, GA, USA; 8 The Center for Comprehensive Access and Delivery Research and Evaluation (CADRE) at the Iowa City VA Healthcare System, University of Iowa Carver College of Medicine, Iowa City, IA, USA; 9 Department of Pharmacy Services, Brigham and Women’s Hospital, Boston, MA, USA; 10 Sunnybrook Health Sciences Centre, University of Toronto, Toronto, Canada; 11 American Society of Health-System Pharmacists Research and Education Foundation, Bethesda, MD, USA; 12 American Association of Critical Care Nurses, Aliso Viejo, CA, USA; 13 Inpatient Clinician Educator Service, Massachusetts General Hospital; Harvard Medical School, Boston, MA, USA; 14 Division of Hospital Medicine, Northwestern University Feinberg School of Medicine, Chicago, IL, USA; 15 BWH Hospitalist Service, Division of General Medicine, Brigham and Women’s Hospital; Harvard Medical School, Boston, MA, USA

**Keywords:** Medication reconciliation, Hospitalization, Quality improvement, Care transitions

## Abstract

**Background:**

Unresolved medication discrepancies during hospitalization can contribute to adverse drug events, resulting in patient harm. Discrepancies can be reduced by performing medication reconciliation; however, effective implementation of medication reconciliation has proven to be challenging. The goals of the Multi-Center Medication Reconciliation Quality Improvement Study (MARQUIS) are to operationalize best practices for inpatient medication reconciliation, test their effect on potentially harmful unintentional medication discrepancies, and understand barriers and facilitators of successful implementation.

**Methods:**

Six U.S. hospitals are participating in this quality improvement mentored implementation study. Each hospital has collected baseline data on the primary outcome: the number of potentially harmful unintentional medication discrepancies per patient, as determined by a trained on-site pharmacist taking a “gold standard” medication history. With the guidance of their mentors, each site has also begun to implement one or more of 11 best practices to improve medication reconciliation. To understand the effect of the implemented interventions on hospital staff and culture, we are performing mixed methods program evaluation including surveys, interviews, and focus groups of front line staff and hospital leaders.

**Discussion:**

At baseline the number of unintentional medication discrepancies in admission and discharge orders per patient varies by site from 2.35 to 4.67 (mean=3.35). Most discrepancies are due to history errors (mean 2.12 per patient) as opposed to reconciliation errors (mean 1.23 per patient). Potentially harmful medication discrepancies averages 0.45 per patient and varies by site from 0.13 to 0.82 per patient. We discuss several barriers to implementation encountered thus far. In the end, we anticipate that MARQUIS tools and lessons learned have the potential to decrease medication discrepancies and improve patient outcomes.

**Trial registration:**

Clinicaltrials.gov identifier NCT01337063

## Background

One of the most prevalent hazards facing hospitalized patients is unintentional medication discrepancies, i.e. unexplained differences in documented medication regimens across different sites of care [1,2]. Unresolved medication discrepancies can contribute to adverse drug events (ADEs), resulting in patient harm [3,4]. Nearly two-thirds of inpatients have at least one unexplained discrepancy in their admission medication history, and some studies found up to 3 medication discrepancies per patient [5-7]. Such medication discrepancies are either caused by history errors (i.e., errors in determining a patient’s preadmission medication list) or reconciliation errors (i.e., errors in orders despite accurate medication histories) [3,4].

One way to minimize medication discrepancies and improve patient safety is to perform high quality medication reconciliation, defined as the process of identifying the most accurate list of all medications a patient is taking and using this list to provide correct medications for patients anywhere within the healthcare system [8,9]. Since 2005 The Joint Commission (TJC) has required U.S. hospitals to conduct medication reconciliation on admission, upon transfer, and at discharge [10]. Additionally, the World Health Organization has encouraged all member states to implement medication reconciliation at care transitions [11]. When tested, hospital-based medication reconciliation interventions have consistently demonstrated reductions in medication discrepancies, though effects on more distal outcomes such as readmission have been less consistent and limited by study size [12,13]. Yet, one study at two large urban academic hospitals found that general medical inpatients averaged more than one potentially harmful discrepancy in either admission or discharge medication orders despite documented completion of medication reconciliation [14].

Though medication reconciliation practices are required at care transitions throughout hospitalization, implementation has been challenging for many hospitals because it often involves a dramatic change in work processes and additional tasks for busy clinicians. Furthermore, the implementation of medication reconciliation interventions varies widely across hospitals, and hospitals need clearer guidance on which interventions are more likely to be successful in their local environment [15]. Moreover, it has been relatively easy for hospitals to document compliance with medication reconciliation processes to meet national and international standards without demonstrating that medication safety has actually improved. To identify and address the barriers to implementing medication reconciliation, an Agency for Healthcare Research and Quality (AHRQ)-funded conference organized by the Society of Hospital Medicine (SHM) in 2009 brought together 36 key stakeholders from 20 organizations representing healthcare policy, patient safety, regulatory, technology, and consumer and medical professional groups. The conference yielded a White Paper with recommendations, including a call for further research [16]. To address the latter, SHM subsequently received funding from AHRQ to conduct the Multi-Center Medication Reconciliation Quality Improvement Study (MARQUIS; clinicaltrials.gov identifier NCT01337063).

The specific aims of MARQUIS are to:

1. Develop a toolkit consolidating the best practices for medication reconciliation, based on the strongest evidence available.

2. Conduct a multi-site mentored quality improvement (QI) study in which each site adapts the tools for its own environment and implements them.

3. Assess the effects of medication reconciliation QI interventions on unintentional medication discrepancies with potential for patient harm.

4. Conduct rigorous program evaluation to determine the most important components of a medication reconciliation program and how best to implement them.

This paper describes the design and early methodological lessons learned from MARQUIS, an example of real-world, rigorous, mixed methods QI research. It is our hope that the design of the study, rationale for that design, and early experiences will be useful for other medication safety efforts, as well as for QI and patient safety research in general.

## Methods

### Conceptual framework

**Figure 1:**
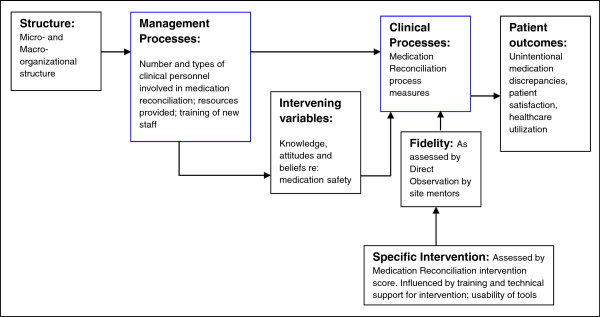
Conceptual framework for MARQUIS.

The MARQUIS conceptual framework is based on Brown and Lilford’s model for evaluating patient safety interventions, which is an adaptation of Donabedian’s “structure—process—outcome” model [17-21]. The model distinguishes interventions that focus on management processes (e.g., provider training) from those that focus on clinical processes (e.g., tools supporting medication list comparisons across care transitions). MARQUIS involves both types of interventions, focusing primarily on the latter. To better understand why interventions succeed or fail, we assess contextual factors (i.e., micro- and macro-organizational structure and existing management processes) and intervening variables (e.g., team climate, safety culture, and knowledge of medication safety principles, Figure 1). Also important to understanding whether interventions succeed or fail is intervention fidelity, or the faithfulness with which the intervention is performed, which can be influenced by how usable the tools are and the degree of training and support given to front-line clinicians (i.e., items at the level of intervention in Figure 1). These are measured as an “intervention score” updated monthly to assess what toolkit components have been adopted and how the state of medication reconciliation changes over time (Table 1). Along with the MARQUIS intervention itself, each of the contextual factors may affect the patient-level outcomes being assessed, such as unintentional medication discrepancies, patient satisfaction, and healthcare utilization (i.e., items to the right of the intervention, Figure 1).

**Table 1:** Intervention components and scoring system

**Toolkit component**	**Standardization by function**	**Scoring system for analysis**
Definition of Medication Reconciliation	Definition exists, is widely disseminated and can be articulated by staff involved in the medication reconciliation process	0-24 points in 8-point increments, depending on whether definition exists, is widely implemented, and can be articulated by >80% of staff involved in the medication reconciliation process
Assigning roles and responsibilities to clinical personnel	Roles and responsibilities are well defined for each phase of medication reconciliation and can be articulated by staff involved in the medication reconciliation process; process owner (e.g., attending physician) is well defined and known by those who own the process	0-12 points in 4-point increments, depending on whether roles are well defined, defined for each phase of the medication reconciliation process, and can be articulated by >80% of staff
		0–12 points in 4 point increments, depending on whether process owner is well defined and what proportion of staff in that role can articulate that they in fact own the process
Improving access to preadmission medication sources	All sites improve exchange of medication information across settings, e.g., community pharmacy prescription information, outpatient medication lists, and inpatient discharge medication orders to all clinical settings	0-24 points in 6 point increments for **electronic** access to outpatient pharmacy information, access to outpatient medications, access to discharge medication orders from prior hospitalizations, and access to patient personal health records.
		(can get up to 12 points if have facilitated paper access to these sources)
Encouraging patient-owned medication lists	All sites develop (on paper or	0-24 points in 6 point increments, depending on whether a standard medication form exists, to what extent patients use it, whether a system is in place to keep it updated, and whether the form is universally accessible
	electronically) a universal instrument to capture the current medication list, based on steering committee guidelines	
Educating providers on how to take a best possible medication history	Providers receive training in taking a best possible medication history, receive feedback on their skills, and have time to perform it well	0-12 points in 4 point increments, depending on whether clinicians are trained to take a medication history, whether time is available to take an adequate history in >80% of patients, and what portion of the staff have ever received feedback in their history taking
Implementing discharge counseling that includes patient education and teach back	Providers counsel patients regarding discharge medications using a standard script that accommodates patients with low health literacy	0-12 points in 4 point increments, depending on whether a standard script is available for discharge counseling, whether health literacy tools are used, and whether >80% of staff is trained in discharge counseling, including patient centered communication
Identifying patients as high vs. low-intermediate risk by stratification	Sites use established risk factors to identify patients at high risk for medication errors, and patient risk drives the type of intervention received	0-24 points available by calculating the product of the two below areas:
		0–4 points available depending on whether there is a standard tool available to identify high risk patients and is used in >80% of patients
		0–6 points available depending on whether the tool drives the intervention intensity, and >80% of eligible patients receive the high-intensity intervention
Implementing intense vs. standard bundle	High-risk patients receive a high-intensity medication reconciliation bundle by providers who are trained and have time to carry it out	0-24 points in 6 point increments depending on whether definition exists for standard and intense intervention, is embraced widely, staff are well trained, and are given adequate time to carry out the intensive bundle in high-risk patients
Implementing and improving electronic medication reconciliation applications where possible	Where possible, take advantage of electronic health record infrastructure and electronic medication reconciliation products to facilitate bidirectional transfer of medication information across settings, compare regimens across settings, and electronically document the reconciliation process	26 maximum points available based on electronic medication reconciliation tools having the following features: ability to compare various sources of preadmission medication information, access to medication adherence information, ability to document and verify a medication history, facilitation and verification of admission and discharge medication reconciliation, facilitation of admission and discharge order-writing, facilitation of patient/caregiver education, tools to facilitate communication with post-discharge providers, features to improve the reliability of the medication reconciliation process, and tools to identify high risk patients
Implementing components using phased approach	Sites implement medication reconciliation improvements in a phased manor using best practices for continuous quality improvement	0-24 points in 6 point increments, depending on whether a plan exists to modify the intervention over time, to expand the intervention beyond the initial pilot sites, whether a time frame for expansion has been established and if the QI team has all the right personnel
Utilizing social marketing and engaging community resources	Sites identify, cultivate, and improve relationships with community resources such as local or regional QI organizations, dominant local pharmacies and payors, and local public health agencies with a goal of working together to improve patient education, transfer of information, and aligning financial incentives	0-24 points in 3 point increments, depending on usage of community resources and a patient safety advisory board in medication reconciliation, and usage of social marketing techniques with patients and providers

### Toolkit

The MARQUIS toolkit, described elsewhere, [22] synthesizes best practices in medication reconciliation and provides aids to facilitate their implementation. The toolkit components were informed by a systematic review of medication reconciliation interventions, [12] the AHRQ-funded conference of stakeholders,[16] and the work of the MARQUIS investigators and advisory board.

Each toolkit component is framed as a standardized functional goal (e.g., “Improve access to preadmission medication sources”). This approach is ideal for complex QI interventions, [23] allowing sites to: 1) integrate intervention components with their baseline medication reconciliation efforts, information system capabilities, and organizational structures; and 2) add, customize, and iteratively refine the toolkit components and their implementation over time. This approach also improves generalizability, allowing other organizations to apply the lessons learned regardless of their culture or unique circumstances.

While recognizing the importance of flexibility, it was nevertheless important to have some common elements across sites. Thus, each site prioritized the implementation of certain toolkit components based on their potential for improvement and effort required. These included provider education on medication history taking, patient education and teach-back at discharge, patient risk stratification, and more intensive medication reconciliation efforts in high-risk patients.

### Study sites

Six U.S. sites are participating in this study: 3 academic medical centers, 2 community hospitals, and 1 Veterans Affairs hospital. We purposely chose sites that vary in size, academic affiliation, geographic location, and use of health information technology (Table 2). However, all sites had several common features: 1) medication reconciliation was a priority; 2) hospital leadership was committed to making further improvements in the process; 3) an active hospitalist group was engaged in QI; 4) a suitable hospitalist and/or pharmacist clinical champion at each site; and 5) each site planned to use primarily its own resources to pursue this effort.

**Table 2:** Baseline characteristics of participating sites

**Site**	**1**	**2**	**3**	**4**	**5**	**6**
Hospital Type	AMC^1^ / Community	Community	Community	AMC	AMC	VAMC^2^
Region	Northeast	Southeast	Southeast	Midwest	West Coast	Midwest
Setting	Urban	Suburban	Suburban	Urban	Urban	Rural
Number of Beds	653	110	535	600	450	45
Teaching Status	Teaching	Teaching	Non-teaching	Teaching	Teaching	Teaching
Inpatient CPOE^3^	Yes (Cerner)	No (moving to Cerner)	No	Yes (Epic)	No (moving to Epic)	Yes
Medication Reconciliation Software	Yes, integrated with CPOE	No (but yes with Cerner)	Yes	Yes, integrated with CPOE	In progress (yes with Epic/Apex)	Yes, not fully integrated
% patients for whom site has electronic access to ambulatory medication history	50%	0%	<10%	~100%	50%	95%
Clinicians primarily responsible for taking medication histories	Jointly shared by physicians and nurses	Nurses first, then physicians	Pharmacy and nursing	Nurses	Physicians	Residents and PAs
Process of medication reconciliation at discharge	Physicians use electronic tool to reconcile medications	Nurses fill out a reconciliation form, physicians reconcile medications	Physicians reconcile medications using paper form	Physicians/NPs/	Physicians write orders, pharmacists available by request to reconcile medications	Physicians or pharmacists, depending on time of day
				PAs^4^ reconcile discharge medications		

1 Academic medical center.

2 Veterans Affairs medical center.

3 Computerized physician order entry.

4 Nurse practitioners/Physician assistants.

Patient subjects are drawn from the medical and surgical inpatient, non-critical care units of each site, and are included if hospitalized long enough for a “gold-standard” medication history to be obtained by a study pharmacist (i.e., generally more than 24 hours). Institutional Review Board (IRB) approval was obtained from the Partners Healthcare System. In addition, each study site’s IRB reviewed the study: four considered it an exempt QI project, while two sites required informed consent of patients prior to participation. Informed consent has been incorporated into the data collection process at these sites.

### Mentored local implementation

MARQUIS utilizes SHM’s mentored implementation approach [24], providing each site with a hospitalist mentor to facilitate toolkit implementation. Each mentor has QI expertise and performs distance mentoring through monthly calls with the study site’s mentee/clinical champion, based upon the MARQUIS Implementation Guide which explains how to use the toolkit [25]. Each study site also receives two visits from the mentor, important from a QI standpoint (e.g., to maintain institutional support and enthusiasm among the local QI team, and better understand local practices) and from a research standpoint (e.g., to assess intervention fidelity and other barriers and facilitators of implementation). Additionally, SHM provides sites with an assigned lead project manager and research assistants located at SHM headquarters to assist with monitoring progress and collecting and analyzing data. Through the mentored implementation infrastructure, MARQUIS is affordable, adaptable, generalizable, scalable, and feasible for wide dissemination.

At each study site, a local QI team, led by the mentee/clinical champion conducts monthly meetings to oversee intervention implementation and data collection, as well as to address protocol questions and determine the effectiveness of the interventions. Sites can access a central website with additional resources and a listserv. The monthly conference calls with their mentor and ad lib email communications promote a consistent approach across sites [16].

### Outcome assessment

Study outcomes are assessed from 6 months pre-intervention through 21 months post-intervention (Table 3). The primary outcome is the number of potentially harmful unintentional medication discrepancies per patient, determined by a trained on-site pharmacist taking a “gold standard” medication history on a random sample of patients (20–25 per month). This history is then compared to the primary team’s medication history and to admission and discharge orders. For discrepancies in admission or discharge orders not caused by history errors, the pharmacist reviews the medical record for a clinical explanation, and if necessary, talks with the medical team. This allows sites to distinguish unintentional medication discrepancies (i.e., due to reconciliation errors) from intentional medication changes. Physician adjudicators, blinded to the status of intervention implementation, record and categorize unintentional medication discrepancies with respect to: 1) timing (admission vs. discharge); 2) type (omission, additional medication, change in dose, route, frequency, or formulation, or other); 3) reason (history vs. reconciliation error); 4) potential for harm; and, 5) potential severity.

**Table 3:** Primary and secondary patient outcomes

**Outcome**	**Description**
**Primary outcome**	
Unintentional Medication Discrepancies in admission and discharge orders with potential for patient harm	Number of discrepancies per patient with potential for harm
**Process measures**	
Accuracy of preadmission medication history	Proportion with accurate medication histories; number of history errors per patient with potential for harm
Absence of discharge reconciliation errors	Proportion with error-free discharge medication orders; number of discharge reconciliation errors per patient
Preadmission medication history documented within 24 hours of admission	Proportion of cases with on-time preadmission medication history documentation
**Other outcome measures**	
Emergency Department visit or readmission to index hospital within 30 days of discharge	Proportion of patients with ED visit or readmission
Patient Satisfaction on HCAHPS^1^ survey	Global satisfaction score; medication specific score; proportion who responded “usually or always” to medication questions

^1^ Hospital Consumer Assessment of Healthcare Providers and Systems.

Secondary outcomes include accuracy and timeliness of the medical team’s documented admission medication history, absence of discharge reconciliation errors, unplanned healthcare utilization (i.e., readmission or emergency department use), and patient satisfaction. Unplanned healthcare utilization to the same site within 30 days of discharge is determined from hospital records on all eligible patients. We examine patient satisfaction scores on the global satisfaction and the medication specific dimensions from the Hospital Consumer Assessment of Healthcare Providers and Systems (HCAHPS) survey, and will be aggregated by service, unit, and time period as data are available.

Contextual factors are measured using surveys of providers directly involved in the medication reconciliation process. Additionally, we measure intervention fidelity using direct observation during site visits, and evaluate training, support, and other steps offered to improve fidelity. Importantly, the extent of implementation is quantified as an intervention “score” for each toolkit component (Table 1) and factored into the analysis. The score is completed by the clinical champion at each site, informed by surveys administered to front-line clinicians who are directly involved in the medication reconciliation process when necessary.

### Data quality assurance

In an effort to ensure consistency of on-site pharmacist data collection, the research team: 1) conducts monthly phone meetings with on-site pharmacists in which a patient case is reviewed for consistency and all discrepancies discussed; 2) provides on-site pharmacists with an updated ‘frequently asked questions’ (FAQ) document for managing new situations; and, 3) conducts site visits with the research team’s pharmacist to observe data collection processes and provide feedback, including how to improve process efficiency.

To ensure the consistency of the adjudication process, the principal investigator (PI) conducts a quarterly conference call with the sites’ physician adjudicators to discuss cases. In addition, the PI and a co-investigator review 6 cases from each site quarterly and review the results individually with each site’s adjudicators. A FAQ document for adjudicators is updated and redistributed as needed.

### Web-based data center

The study sites utilize a web-based data collection and reporting system built specifically for this study. The system creates HIPAA-compliant de-identified data sets for the coordinating data center and all investigators. The system allows for identification, classification, and adjudication of all discrepancies. Unintentional discrepancies identified by the on-site pharmacist are flagged in the system for physician adjudication. The data center provides detailed reports to trend discrepancies, facilitates uploads of patient-specific administrative data, tracks implementation of intervention components, and provides tools to support mentored implementation. It also provides tracking for patient enrollment compared to monthly targets.

### Statistical analysis

The primary outcome will be analyzed using multivariable Poisson regression, including random effects and clustering of patients by site and treating physician. To account for temporal trends and the varied introduction of interventions by site, we will employ an interrupted time series analysis on all 3,600 patients across the 6 sites, evaluating outcomes monthly for 6 months pre-intervention and 21 months post-implementation [26]. The outcome is assessed as both a change from site-specific baseline temporal trends (i.e., change in slope) and sudden improvement with implementation of the intervention components (i.e., change in y-intercept). If each site has concurrent controls, these can be entered into the model to partially adjust for the effect of concurrent interventions. The model also allows for the detection of iterative refinement of the intervention (i.e., continuous improvement over time), as well as ceiling effects (i.e., lack of continued improvement beyond a certain threshold).

We will assess implementation of each component of the toolkit monthly, using the scoring system on a scale of no adoption to complete adoption for each component (Table 1). Because many of the sites have already implemented pieces of the interventions, their scores often do not start at zero, and scores increase with the implementation of interventions to a maximum score of 318. The scores for each component will be entered into the multivariable model as time-varying covariates, such that we can determine whether implementation of a particular component is correlated with improved outcomes thereafter. This allows us to make inferences about the most important components of the intervention.

### Power and sample size

For a stable estimate of temporal trends, each site’s data collection goal is approximately 22 patients per month, beginning 6 months pre-intervention through 21 months post-intervention. Due to our study design it is impossible to know *a priori* the nature of our post-intervention data, and therefore what our actual power would be to look at the effect of any specific intervention. However, based on prior research, we assumed that the number of medication discrepancies would follow a Poisson distribution and that, in the absence of an intervention, each hospitalized patient would have an average of 1.5 potentially harmful medication discrepancies in admission and discharge orders combined [27]. We also conservatively assumed that an intervention would be implemented at only 1 of 6 sites with 12, not 21, months of follow-up due to delays in planning and phasing in the intervention widely. This would yield data from 133 patients pre-intervention and 266 patients post-intervention. With these estimates and alpha = 0.05, we would have 90% power to detect a reduction in the mean number of medication discrepancies from 1.5 per patient to 1.1 per patient [27].

As sites began to implement the intervention, one methodological issue that arose was the extent to which sites should over-sample data from hospital areas receiving early versions of the intervention. We decided on a 3:1 ratio of intervention to control patients during the intervention period. This allows for concurrent controls during the spread of the intervention, while maintaining an adequate sample of intervention patients to evaluate effects on patients outcomes.

### Program evaluation

We will evaluate the influence of contextual factors, intervention fidelity, and intervening variables on implementation and outcomes using a mixed methods approach (Table 4). Measures of context are gathered using front-line staff and site surveys, direct observation, focus groups and interviews. At baseline, each site completed a site leader survey and front-line staff surveys to provide a semi-quantitative measurement of these issues. After the start of intervention implementation, a qualitative researcher conducts on-site focus groups with front-line staff and the QI team and interviews hospital leadership. At 12 months post-intervention, follow-up interviews are conducted by telephone. For focus groups and interviews, a convenience sample of staff is selected by role and department to ensure broad representation [28].

**Table 4:** Program evaluation

**Outcome**	**Timing**	**Data sources**	**Time required**	**Data collection process**	**Form of analytic variable**
**Intervention assessment**					
Medication Reconciliation Intervention Score	Monthly throughout the intervention	Surveys to site leaders at each site, confirmed by mentor	1 hour for baseline assessment, 15–20 minutes for subsequent assessments	Survey completed in QuesGen	0-24 scale for each facet of medication reconciliation; total score
Front-line staff Surveys to inform medication reconciliation intervention score	As needed throughout the study period as interventions implemented likely to affect results	Surveys completed by stakeholders (separate survey for outpatient clinicians)	10 minutes per survey	Survey administered to all potential stakeholders using on-line survey software	Results used descriptively and to inform Medication Reconciliation intervention score
**Measures of context**					
Macro- and Micro-organizational structure	Prior to intervention	Modification of RAND ICICE organizational survey [29] completed by site leaders with help from administrative/financial personnel	1 hour per site	Survey emailed to respondents	Varies by question type
Safety culture, work climate, and teamwork	Prior to intervention	Modification of AHRQ patient safety culture survey [30] completed by stakeholders (e.g., pharmacists, nurses, physicians)	10 minutes per survey	Survey administered to all potential stakeholders using on-line survey software	Composite frequency of positive responses in each of 10 dimensions of safety
Satisfaction with medication reconciliation process and software, perceptions of errors related to medication reconciliation	Prior to intervention, again post-intervention	Survey completed by stakeholders	5 minutes per survey	As with safety culture	Frequency of positive responses
Job satisfaction and burnout	Prior to intervention, again post-intervention	Surveys [31,32]	5 minutes per survey	As with safety culture	Frequency of positive responses
**Qualitative information**					
Focus Groups	At first site visit	5 focus groups of 6–8 representative stakeholders each, grouped by type, 35 total per site	60-90 minutes	Administered by qualitative researcher	
Individual Interviews	Follow-up phone calls one year after focus groups	One-on-one interviews with champions and 2–3 key opinion leaders per site	30-45 minutes	Administered by phone by qualitative researcher	
**Intervention fidelity**					
Intervention Fidelity	At each site visit	Direct observation of medication reconciliation process	6 hours at each visit (over two days)	Mentor	Mean percent completion of each process component; process fidelity scale (1–4)

Intervention fidelity is assessed by direct, semi-structured observation of the site’s medication reconciliation process by their mentor during site visits at 3 and 12 months after implementation of the intervention. The observation protocol evaluates five steps of the medication reconciliation process: taking an admission medication history, identifying high risk patients to receive a high intensity medication reconciliation intervention, performing discharge medication reconciliation, performing discharge medication counseling using the teach back method, and forwarding the discharge medication list to the next provider of care after discharge. The mentor observes the actual process to identify if the intervention is being implemented as designed (content fidelity) and rates how well it was performed on a 1 to 4 scale (process fidelity) [33]. The observation forms also allow documentation of systems issues that impact the medication reconciliation process based on the Systems Engineering Initiative for Patient Safety (SEIPS) model’s [34] 5 domains: people, technology/tools, tasks, organization, and environment. Mentors received group training on how to assess fidelity, using a coding manual with standardized examples and training from a human factors expert. Mentors share feedback about the direct observations during the site visit with the site leader and QI team.

Intervening variables (i.e., in determining intervention fidelity) are assessed from surveys of front-line staff pre- and post-intervention. Topics include the quality of education and training received in the intervention (e.g., in taking medication histories), degree of input into intervention design, and adequacy of staffing and time to complete medication reconciliation processes. Other barriers and facilitators of intervention implementation are determined from structured and open-ended questions regarding the front-line staff’s opinions of the medication reconciliation process and its perceived impact on patient care.

### Study timeline

**Figure 2:**
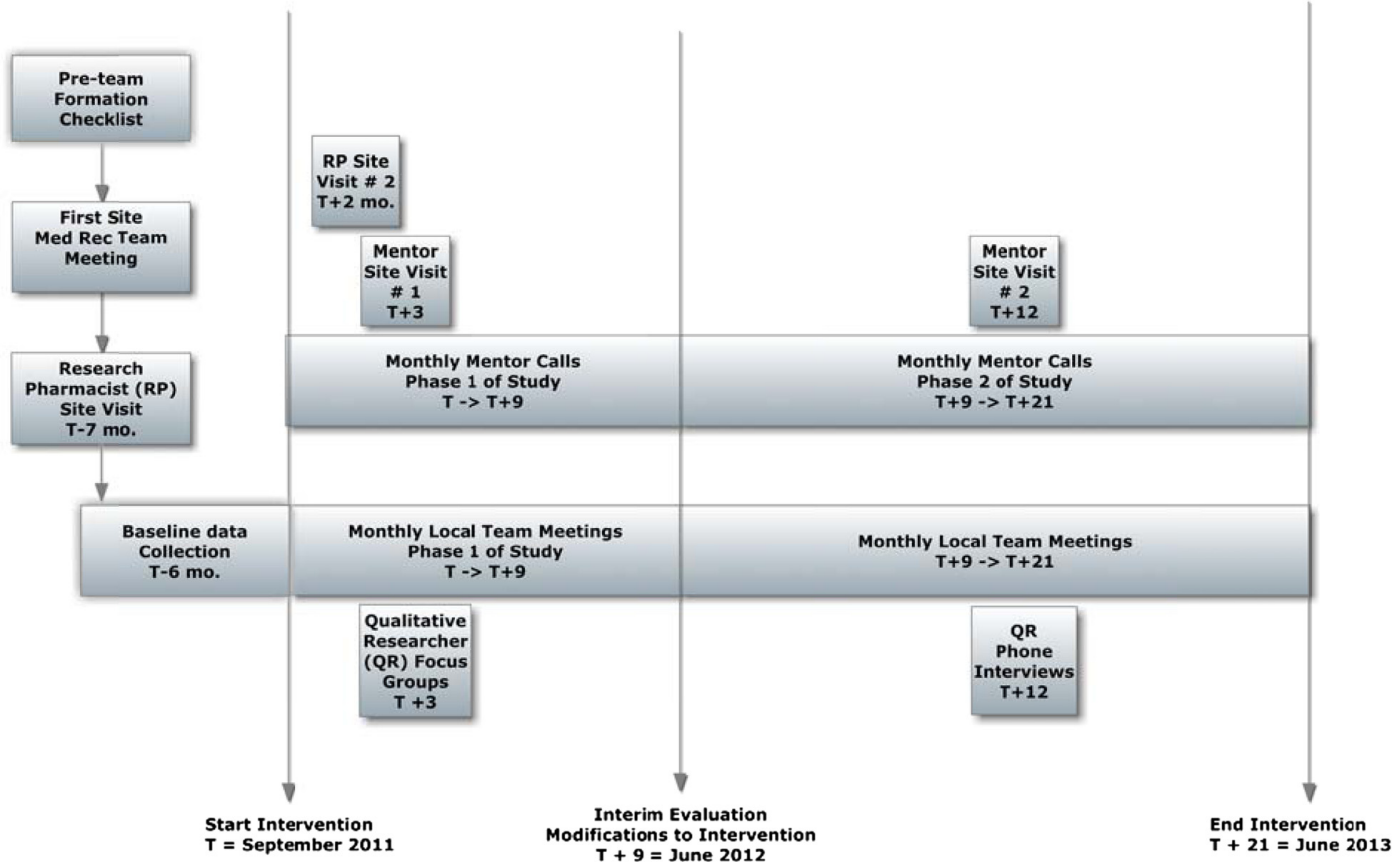
MARQUIS study timeline.

Figure 2 outlines the timeline of the study. The study’s three critical time points are: intervention start, interim evaluation with iterative refinement of the intervention and a second draft of the implementation guide 9 months after the start, and end of the intervention after 21 months.

## Discussion

Baseline data collection began March 2011 and was completed August 2012. Over 1,000 patients have been enrolled to date across the 6 sites, of which 980 have data entered in a centralized database (Table 5). Of these, preliminary analyses show 844 patients have had discrepancies adjudicated for potential harm. The total number of unintentional medication discrepancies in admission and discharge orders per patient varies by site from 2.35 to 4.67 (mean = 3.35). Consistent with prior research, most discrepancies are due to history errors (mean 2.12 per patient) as opposed to reconciliation errors (mean 1.23 per patient) [14]. The number of potentially harmful medication discrepancies averages 0.45 per patient and varies by site from 0.13 to 0.82 per patient. In comparison, in prior studies by Schnipper et al. potentially harmful medication discrepancies began at 1.44 per patient, then decreased to 1.05 and then 0.32 per patient with successive versions of medication reconciliation interventions [27,35].

**Table 5:** Baseline results

	**All sites**	**A**	**B**	**C**	**D**	**E**	**F**
	**(N=927)**	**(n=313)**	**(n=360)**	**(n=124)**	**(n=150)**	**(n=82)**	**(n=22)**
**Total discrepancies per patient (history and reconciliation): admission and discharge**	**3.77**	**4.52**	**2.43**	**2.91**	**3.27**	**3.16**	**1.73**
Total discrepancies at admission	1.72	2.16	1.11	1.02	1.49	1.35	0.73
Total discrepancies at discharge	2.05	2.36	1.33	1.89	1.79	1.80	1.00
**History discrepancies: admission and discharge**	**2.39**	**3.36**	**1.98**	**1.91**	**0.66**	**0.87**	**2.59**
History discrepancies: admission	0.97	1.34	0.88	0.44	0.27	0.65	1.91
History discrepancies: discharge	1.41	2.02	1.10	1.47	0.39	0.22	0.68
**Reconciliation discrepancies: admission and discharge**	**1.38**	**1.16**	**0.44**	**1.00**	**2.61**	**2.29**	**2.59**
Reconciliation discrepancies: admission	0.75	0.82	0.22	0.58	1.22	0.71	1.91
Reconciliation discrepancies: discharge	0.64	0.35	0.23	0.42	1.39	1.59	0.68
Adjudicated results							
**Number of potentially harmful discrepancies per patient**^**1**^**: total**	**0.46**	**0.26**	**0.45**	**0.67**	**0.70**	**0.82**	**0.36**
Potentially harmful discrepancies: admission	0.16	0.12	0.17	0.17	0.15	0.29	0.09
Potentially harmful discrepancies: discharge	0.30	0.14	0.28	0.50	0.55	0.53	0.27
Potential severity: admission	0.12	0.10	0.12	0.14	0.13	0.23	0.00
Significant							
Serious^2^	0.04	0.03	0.05	0.03	0.03	0.06	0.09
Potential severity: discharge	0.23	0.13	0.21	0.44	0.40	0.32	0.18
Significant							
Serious^2^	0.07	0.01	0.07	0.06	0.15	0.21	0.09

^1^ Greater than 50% chance that the medication discrepancy identified had potential to harm patient.

^2^ There were no life-threatening discrepancies.

Challenges have arisen during the initial implementation. From a research perspective, these included delays in signing data use agreements, IRB requirement for patient consent at some sites, delays in obtaining IRB approval, and staffing challenges, all of which led to delays in completing baseline data collection. Another challenge was achieving adequate response rates from front-line surveys. In the end, we asked each site to identify a select group of clinicians likely to complete surveys while still representative of the locations and provider types involved in the medication reconciliation process, sacrificing some generalizability for better rates (and thus internal validity). Operationally, rather than create a distinct “version 2” of the intervention, we chose to iteratively refine and add to our toolkit as sites and mentors have identified specific needs, although we do plan to develop a distinct “second edition” of the implementation guide.

To date, initial site visits have been conducted at 3 of the 6 sites within 4 months of start of the intervention implementation. Feedback after mentor and qualitative researcher visits were shown to be more valuable at sites that were further along with intervention implementation. This timing allowed for more feedback on barriers and facilitators to implementation from focus group participants intimately involved in the intervention, and allowed for richer data collection on intervention fidelity, creating more detailed feedback to sites on how to improve their interventions going forward. On the other hand, because site visits also enhanced the visibility and institutional support of the project at the sites, for those sites that were struggling with implementation, the decision was made to conduct these visits on time anyway, trading some data loss regarding intervention fidelity for gains in local support.

MARQUIS seeks to improve medication safety at participating hospitals, while rigorously studying the implementation of a best practices toolkit and contextual factors that may influence outcomes. As such, the study offers one approach to conducting rigorous, “real world” QI research in which we hope to understand: 1) the most important components of the intervention; 2) reasons for success or failure; and, 3) barriers and facilitators of implementation. MARQUIS also attempts to balance the criticism for a more case-based approach to QI research with more rigorous outcome assessment that adequately adjusts for potential confounders.

Importantly, MARQUIS does not provide sites with resources for the intervention and only a small stipend for data collection, similar to QI efforts at most hospitals. This lowered the cost of the study and also makes it more generalizable since other sites wishing to adopt the intervention toolkit most likely would not receive external resources for implementation. Nevertheless, this also makes sites more vulnerable to resource constraints and changes in leadership or institutional priorities, in particular during the lag time between applying for funding and beginning the intervention. As hospitals are increasingly challenged to conserve resources, projects like MARQUIS are more likely to succeed if medication safety is a consistent priority or if a favorable return on investment is anticipated. To address the latter, we have provided sites with business plans on making the business case for medication reconciliation, which is available online in the MARQUIS implementation guide.

Other challenges reflect the need to balance the needs of QI work with research, such as the length of pre-intervention data collection (shorter for QI, longer for research) and the optimal timing of site visits (earlier for QI to enlist institutional support, later for research to best assess intervention fidelity).

Despite these challenges, our use of a mentored implementation model makes MARQUIS a generalizable approach to studying the improvement of complex processes like inpatient medication reconciliation. If the intervention is shown to be successful, mentored implementation resources could easily be scaled up. Using a refined version of the tools and implementation lessons, MARQUIS holds promise to provide a large impact on medication safety during transitions in care across many hospitals.

## Abbreviations

ADEs: Adverse drug events; AHRQ: Agency for healthcare research and quality; AMC: Academic medical center; APNs: Advanced practice nurses; CPOE: Computerized physician order entry; FAQ: Frequently asked questions; HCAHPS: Hospital consumer assessment of healthcare providers and systems; IRB: Institutional review board; MARQUIS: Multicenter medication reconciliation quality improvement study; NPs/PAs: Nurse practitioners/Physician assistants; PI: Primary investigator; QI: Quality improvement; SHM: Society of hospital medicine; TJC: The joint commission; VAMC: Veterans affairs medical center.

## Competing interests

Dr. Kripalani is a consultant to and holds equity in PictureRx, LLC. The terms of this agreement were reviewed and approved by Vanderbilt University in accordance with its conflict of interest policies. Dr. Schnipper is a consultant to QuantiaMD, for whom he helps create educational tools for providers and patients regarding medication safety; these tools are not part of MARQUIS. Dr. Schnipper is also principal investigator of an investigator-initiated study of interventions to improve transitions in care in patients with Diabetes. The terms of these agreements were reviewed and approved by Partners HealthCare and Harvard Medical School in accordance with its conflict of interest policies. All other authors have no competing interests.

## Authors’ contributions

JLS, SK, TBW, JS, and PJK designed the study. EE, DJC, DH, JLG, and MVW are members of the advisory board and provided input throughout the study to assist in refining the process. AHS and JLS drafted the manuscript. All authors read, made significant contributions, and approved the final manuscript.

## Pre-publication history

The pre-publication history for this paper can be accessed here:

http://www.biomedcentral.com/1472-6963/13/230/prepub

## References

[B1] Coleman EA Smith JD Raha D Min SJ Posthospital medication discrepancies: prevalence and contributing factors Arch Intern Med 2005 165 1842 1847 10.1001/archinte.165.16.1842 16157827

[B2] Smith JD Coleman EA Min SJ A new tool for identifying discrepancies in postacute medications for community-dwelling older adults Am J Geriatr Pharmacother 2004 2 141 147 10.1016/S1543-5946(04)90019-0 15555490

[B3] Cornish PL Knowles SR Marchesano R Tam V Shadowitz S Juurlink DN Etchells EE Unintended medication discrepancies at the time of hospital admission Arch Intern Med 2005 165 424 429 10.1001/archinte.165.4.424 15738372

[B4] Schnipper JL Kirwin JL Cotugno MC Wahlstrom SA Brown BA Tarvin E Kachalia A Horng M Roy CL McKean SC Bates DW Role of pharmacist counseling in preventing adverse drug events after hospitalization Arch Intern Med 2006 166 565 571 10.1001/archinte.166.5.565 16534045

[B5] Tam VC Knowles SR Cornish PL Fine N Marchesano R Etchells EE Frequency, type and clinical importance of medication history errors at admission to hospital: a systematic review CMAJ Canadian Medical Association Journal 2005 173 510 515 10.1503/cmaj.045311 PMC1188190 16129874

[B6] Climente-Marti M Garcia-Manon ER Artero-Mora A Jimenez-Torres NV Potential risk of medication discrepancies and reconciliation errors at admission and discharge from an inpatient medical service Ann Pharmacother 2010 44 1747 1754 10.1345/aph.1P184 20923946

[B7] Chan AH Garratt E Lawrence B Turnbull N Pratapsingh P Black PN Effect of education on the recording of medicines on admission to hospital J Gen Intern Med 2010 25 537 542 10.1007/s11606-010-1317-x 20237959 PMC2869408

[B8] Medication reconciliation review http://www.ihi.org/knowledge/Pages/Tools/MedicationReconciliationReview.aspx

[B9] Shekelle PG Pronovost PJ Wachter RM McDonald KM Schoelles K Dy SM Shojania K Reston JT Adams AS Angood PB The Top patient safety strategies that Can Be encouraged for adoption Now Ann Intern Med 2013 158 365 368 10.7326/0003-4819-158-5-201303051-00001 23460091

[B10] Van Houtven CH Jeffreys AS Coffman CJ Home health care and patterns of subsequent VA and medicare health care utilization for veterans Gerontologist 2008 48 668 678 10.1093/geront/48.5.668 18981283

[B11] WHO Collaborating Centre for Patient Safety Solutions Assuring medication accuracy at transitions in care Book Assuring medication accuracy at transitions in care 2007 vol. 1 City: World Health Organization

[B12] Mueller SK Sponsler KC Kripalani S Schnipper JL Hospital-based medication reconciliation practices: a systematic review Arch Intern Med 2012 172 1057 1069 10.1001/archinternmed.2012.2246 22733210 PMC3575731

[B13] Koehler BE Richter KM Youngblood L Cohen BA Prengler ID Cheng D Masica AL Reduction of 30-day postdischarge hospital readmission or emergency department (ED) visit rates in high-risk elderly medical patients through delivery of a targeted care bundle J Hosp Med 2009 4 211 218 10.1002/jhm.427 19388074

[B14] Pippins JR Gandhi TK Hamann C Ndumele CD Labonville SA Diedrichsen EK Carty MG Karson AS Bhan I Coley CM Classifying and predicting errors of inpatient medication reconciliation J Gen Intern Med 2008 23 1414 1422 10.1007/s11606-008-0687-9 18563493 PMC2518028

[B15] Kaboli PJ Fernandes O Medication reconciliation: moving forward Arch Intern Med 2012 172 1069 1070 10.1001/archinternmed.2012.2667 22733283

[B16] Greenwald JL Halasyamani L Greene J LaCivita C Stucky E Benjamin B Reid W Griffin FA Vaida AJ Williams MV Making inpatient medication reconciliation patient centered, clinically relevant and implementable: a consensus statement on key principles and necessary first steps J Hosp Med 2010 5 477 485 10.1002/jhm.849 20945473

[B17] Brown C Hofer T Johal A Thomson R Nicholl J Franklin BD Lilford RJ An epistemology of patient safety research: a framework for study design and interpretation. Part 1. Conceptualising and developing interventions Qual Saf Health Care 2008 17 158 162 10.1136/qshc.2007.023630 18519620

[B18] Brown C Hofer T Johal A Thomson R Nicholl J Franklin BD Lilford RJ An epistemology of patient safety research: a framework for study design and interpretation. Part 2. Study design Qual Saf Health Care 2008 17 163 169 10.1136/qshc.2007.023648 18519621

[B19] Brown C Hofer T Johal A Thomson R Nicholl J Franklin BD Lilford RJ An epistemology of patient safety research: a framework for study design and interpretation. Part 3. End points and measurement Qual Saf Health Care 2008 17 170 177 10.1136/qshc.2007.023655 18519622

[B20] Brown C Hofer T Johal A Thomson R Nicholl J Franklin BD Lilford RJ An epistemology of patient safety research: a framework for study design and interpretation. Part 4. One size does not fit all Qual Saf Health Care 2008 17 178 181 10.1136/qshc.2007.023663 18519623

[B21] Donabedian A Griffith JR Explorations in quality assessment and monitoring The definition of quality and approaches to it assessment 1980 Washington, DC: Health Administration Press 4 163

[B22] Mueller SK Kripalani S Stein J Kaboli P Wetterneck TB Salanitro AH Labonville S Etchells E Hanson D Williams MV Development of a toolkit to disseminate best practices in inpatient medication reconciliation JtComm J Qual Patient Saf 2013 In press 10.1016/s1553-7250(13)39051-5 PMC11110895 23991510

[B23] Hawe P Shiell A Riley T Complex interventions: how "out of control" can a randomised controlled trial be? BMJ 2004 328 1561 1563 10.1136/bmj.328.7455.1561 15217878 PMC437159

[B24] Maynard GA Budnitz TL Nickel WK Greenwald JL Kerr KM Miller JA Resnic JN Rogers KM Schnipper JL Stein JM 2011 John M. Eisenberg patient safety and quality awards. Mentored implementation: building leaders and achieving results through a collaborative improvement model. Innovation in patient safety and quality at the national level Jt Comm J Qual Patient Saf 2012 38 301 310 22852190 10.1016/s1553-7250(12)38040-9

[B25] Schnipper JL Schnipper JL MARQUIS implementation manual: a guide for medication reconciliation quality improvement Book MARQUIS implementation manual: a guide for medication reconciliation quality improvement 2011 City: Society of Hospital Medicine

[B26] McDowall D McCleary R Meidinger EE Hay RA Interrupted time series analysis 1980 Thousand Oaks: SAGE

[B27] Schnipper JL Hamann C Ndumele CD Liang CL Carty MG Karson AS Bhan I Coley CM Poon E Turchin A Effect of an electronic medication reconciliation application and process redesign on potential adverse drug events: a cluster-randomized trial Arch Intern Med 2009 169 771 780 10.1001/archinternmed.2009.51 19398689

[B28] Kuper A Lingard L Levinson W Critically appraising qualitative research BMJ 2008 337 a1035 10.1136/bmj.a1035 18687726

[B29] RAND ICICE organization characteristics survey for ICICE contact 1999 Santa Monica, CA

[B30] Agency for Healthcare Research and Quality Hospital survey on patient safety culturehttp://www.ahrq.gov/professionals/quality-patient-safety/patientsafetyculture/hospital/index.html. Accessed January 10, 2010

[B31] Quinn R Seashore S Kahn R Mangion T Cambell D Staines G McCullough M Survey of working conditions: final report on univariate and bivariate tables Book survey of working conditions: final report on univariate and bivariate tables 1971 City: Government Printing Office

[B32] Wetterneck TB Linzer M McMurray JE Douglas J Schwartz MD Bigby J Gerrity MS Pathman DE Karlson D Rhodes E Worklife and satisfaction of general internists Arch Intern Med 2002 162 649 656 10.1001/archinte.162.6.649 11911718

[B33] Dumas JE Lynch AM Laughlin JE Phillips Smith E Prinz RJ Promoting intervention fidelity. Conceptual issues, methods, and preliminary results from the EARLY ALLIANCE prevention trial Am J Prev Med 2001 20 38 47 10.1016/S0749-3797(00)00272-5 11146259

[B34] Carayon P Hundt AS Alvarado CJ Springman SR Ayoub P Patient safety in outpatient surgery: the viewpoint of the healthcare providers Ergonomics 2006 49 470 485 10.1080/00140130600568717 16717005

[B35] Schnipper J Assessment of improvements to IT-based medication reconciliation Book Assessment of improvements to IT-based medication reconciliation 2010 City: Society of Hospital Medicine

